# Immune Checkpoint Inhibitors in Acute Myeloid Leukemia: A Meta-Analysis

**DOI:** 10.3389/fonc.2022.882531

**Published:** 2022-04-21

**Authors:** Marina Gómez-Llobell, Andrés Peleteiro Raíndo, Jose Climent Medina, Ignacio Gómez Centurión, Adrián Mosquera Orgueira

**Affiliations:** ^1^ Hematology Department, Medical University General Hospital Gregorio Marañon, Madrid, Spain; ^2^ Health Research Institute of Santiago de Compostela (IDIS), Santiago de Compostela, Spain; ^3^ Division of Hematology, Complexo Hospitalario Universitario de Santiago de Compostela (CHUS) University Hospital of Santiago de Compostela (SERGAS), Department of Hematology, Santiago de Compostela, Spain; ^4^ A&P Lifescience Advisors, Madrid, Spain

**Keywords:** acute myeloid leukemia (AML), immune checkpoint inhibitors (ICIs), meta-analysis, Anti-PD1, Anti-CTLA-4, Anti-TIM-3, Anti-CD47, Anti-CD70

## Abstract

**Background:**

Experience with immune checkpoint inhibitors (ICIs) in the treatment of acute myeloid leukemia (AML) is still limited and based on early clinical trials, with no reported randomized clinical data. In this study, we reviewed the available evidence on the use of ICIs, either in monotherapy or in combination with other treatments, in different AML settings, including newly diagnosed AML, relapsed or refractory (R/R) AML and maintenance treatment after allogeneic-HSCT (allo-HSCT).

**Materials and Methods:**

A systematic literature review was conducted using PubMed electronic database as primary source to identify the studies involving immune checkpoint inhibitors in first-line and R/R AML. We recorded Overall Response (ORR), Complete Response (CR) and Complete Response with incomplete count recovery (CRi) rates, overall survival (OS) and immune-related adverse events ≥ grade 3 (irAEs). Hereafter, we analyzed the overall profile of these ICIs by performing a meta-analysis of the reported outcomes.

**Results:**

A total of 13 studies were identified where ICI was used in patients with AML. ORR across these studies was 42% (IC95%, 31% - 54%) and CR/CRi was 33% (IC95%, 22%-45%). Efficacy was also assessed considering the AML setting (first-line vs. relapsed/refractory) and results pointed to higher response rates in first-line, compared to R/R. Mean overall survival was 8.9 months [median 8 months, (IC95%, 3.9 - 15.5)]. Differences between first line and R/R settings were observed, since average overall survival in first line was 12.0 months, duplicating the OS in R/R which was 7.3 months. Additionally, the most specific adverse events (AEs) of these therapies are immune-related adverse events (irAEs), derived from their inflammatory effects. Grade ≥3 irAEs rate was low and similar among studies [12% (95%CI 8% - 16%)].

**Conclusion:**

ICIs in combination with intensive chemotherapy, hypomethylating agents or other targeted therapies are gaining interest in the management of hematological malignancies such as AML. However, results obtained from clinical trials are modest and limited by both, the type of design and the clinical trial phase. Hopefully, the prospective study of these therapies in late-stage development could help to identify patients who may benefit from ICI therapy.

## 1 Introduction

### 1.1 AML

Acute myeloid leukemia (AML) is a malignant neoplasm of abnormally or poorly differentiated cells of the hematopoietic system, characterized by clonal evolution and genetic heterogeneity (1, 2). AML is the most frequent type of acute leukemia in adults, with an incidence of 4.3 cases per 100,000 inhabitants per year. In spite of a significant advance in the understanding of the disease, AML diagnosis establishes a poor prognosis, with a 5-year overall survival of 20-30% (3).The identification of cytogenetic abnormalities and recurrent gene mutations has therapeutic and prognostic implications. In this sense, the use of risk stratification systems combining these findings could guide patient treatment and predict outcome.

In the last years, the development of new drugs with different mechanism of action has dramatically changed the treatment landscape of patients with AML. Monoclonal antibodies (gemtuzumab ozogamicin), FLT3 inhibitors (midostaurin, gilteritinib), IDH1 and IDH2 inhibitors (ivosidenib and enasidenib respectively), new formulations of chemotherapy (CPX-351) and the BCL-2 inhibitor venetoclax, have emerged and been incorporated to standard treatment in different settings of patients with newly diagnosed and relapsed/refractory AML. However, immunotherapy still plays a key role in the treatment of patients with AML, with allogeneic hematopoietic stem cell transplantation (allo-HSCT) being the only curative option for most patients. AML cells can dysregulate immune checkpoints and targeting these control points has been proposed as a treatment strategy and led to the development of immune checkpoint inhibitors (ICI). For instance, monoclonal antibody therapy directed against co-inhibitory signals prevents tumor evasion, blocking the inhibition of cytotoxic T cells and allowing their action against the tumor. ICI has shown efficacy in multiple advanced solid tumors, even in some hematological malignancies (4).

**Figure 1:**
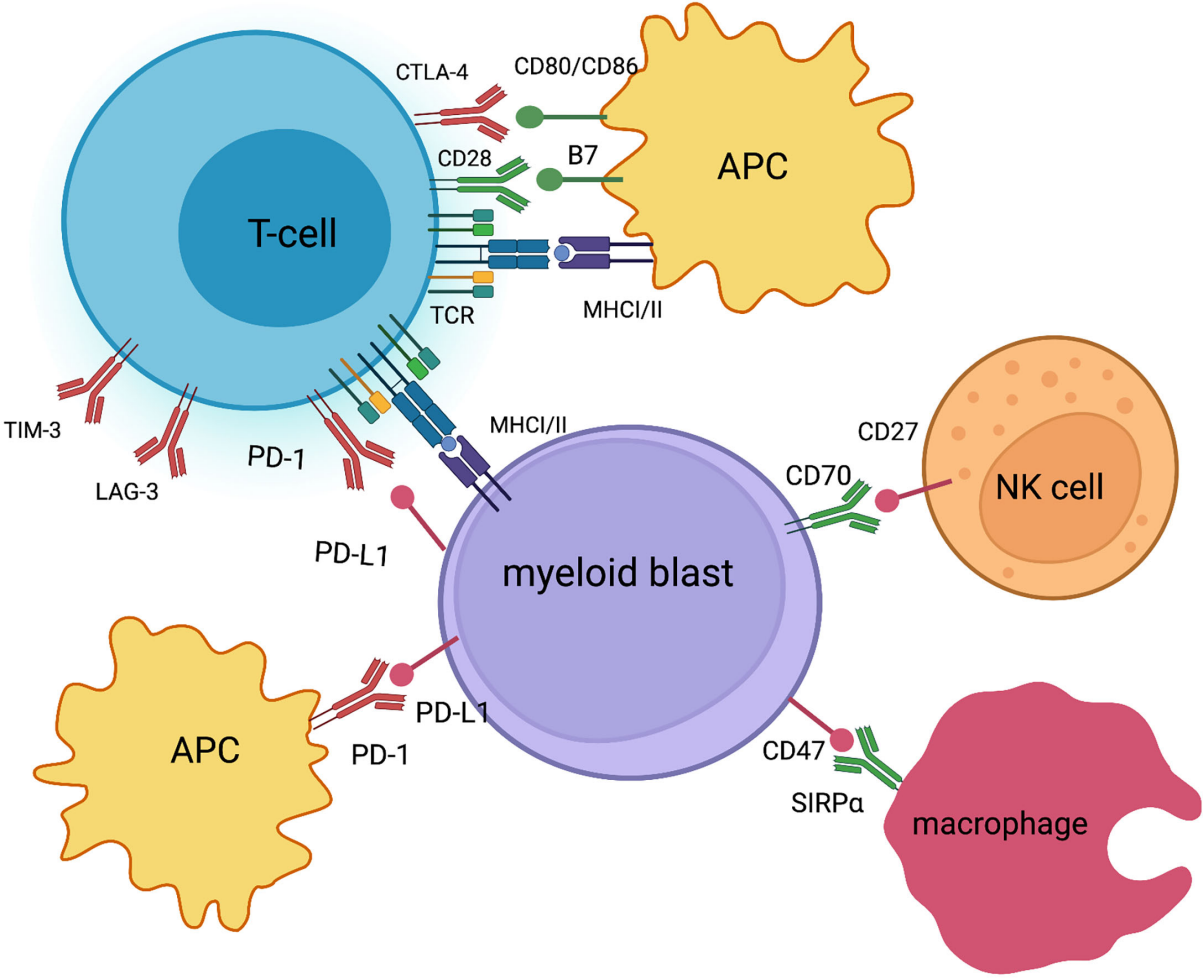
Immune checkpoints in AML co-stimulatory and co-inhibitory signals (created with BioRender.com).

Programmed Death-ligand 1 (PD-1/PD-L1) inhibitors and Cytotoxic T-Lymphocyte Antigen 4 (CTLA-4) inhibitors are the types of ICI which have demonstrated to be safe and effective in different types of cancer, resulting in multiple approvals by drug administrations in solid tumors and some lymphoid malignancies (5). In addition, ICIs have been explored in monotherapy and in combination with other ICIs, with other immunotherapy strategies or in combination with chemotherapy in patients with AML and myelodysplastic syndromes (MDS) (6). It is appropriate to highlight that PD-1 inhibitors are currently the most developed ICI in patients with AML, including pidilizumab, nivolumab, pembrolizumab, durvalumab, and atezolizumab. Increased expression of PD-1 in blasts and in T cells has been documented in a subset of patients with AML and MDS, especially in R/R patients, and is associated with a poor prognosis. Additionally, a benefit of this therapy has been observed in patients with increased expression of PD-1 (7).Moreover, promising results have been observed with ipilimumab, a CTLA-4 inhibitor, in patients with high-risk AML and MDS, including patients with a relapse after allo-HSCT (8). Finally, new T-cell checkpoints, including TIM-3, LAG-3 and the CD47 macrophage checkpoint are being studied in patients with AML (9) (**Figure 1**).

The combination of the ICI together with standard of care treatment in AML is being explored in various trials. Hypomethylating agents (HMAs) are used in AML due to the deregulation of epigenetic regulator genes (DNMT3A, TET2). Consequently, treatments based on HMAs inhibit the action of methyltransferase, leading to restoration of gene expression. This process can lead to an antitumor advantage in terms of the expression of tumor suppressor genes and activation of the immune system. It has been proposed that one of the mechanisms of resistance to HMAs is related to a dose-dependent regulation of the checkpoint of PD-1/PD-L1 in MDS/AML patients (10). On the other hand, the use of intensive cytotoxic chemotherapy induces DNA damage, which could activate the immune system. The release of tumor antigens after cytotoxic chemotherapy could also stimulate a useful immune response against AML. For these reasons, trials are under development based on the combination of chemotherapy or HMAs together with PD-1 inhibitors (11).

In this study, we review the available evidence on the use of ICI, either in monotherapy or in combination with other treatments, in different AML settings, including newly diagnosed (ND) AML, relapsed or refractory (R/R) AML and maintenance treatment after allogeneic-HSCT (allo-HSCT), among others.

## 2 Materials and Methods

The primary objective of this systematic review and meta-analysis is to assess the efficacy of immune checkpoint inhibitor-based immunotherapy (ICI) in acute myeloid leukemia (AML), either after first-line chemotherapy with allo-HSCT, in refractory or relapsed disease and in patients who are not candidates for intensive chemotherapy who are treated with hypomethylating agents. Secondary objectives include the assessment of checkpoint inhibitor-mediated immune-mediated toxicities.

### 2.1 Systematic Search and Search Strategies

Selection criteria was established following the PICOS scheme (**Supplementary Material Figure 1** **)**. The search was run through PubMed according to the association of ICIs and AML terms. Particularly, the search keywords used included leukemia, acute myeloid, acute myelogenous, cancer, neoplasm*, malginanc*, hemato*, haemato*, Anti-PD1, Anti-PD-L1, Anti-CTLA-4, Anti-TIM3, Anti-LAG-3, Anti-CD70 and their drugs names (both commercial and development drug names) in clinical trial development.

After completing the search, all references were downloaded and imported into the Mendeley application and duplicates were removed. References were exported in RIS format, to be then imported by the *ASReview* autonomous learning assistant, which was used to accelerate the process of bibliography review. Furthermore, there have been two types of searches of data sources during the process. First, a general search around a generalized selection of all publications in which there was any kind of association between AML and immunotherapy. Second, an advanced search, including only references that were primary sources of the studies of interest.

### 2.2 Eligibility Criteria and Data Extraction

The inclusion criteria were as follows: randomized and non-randomized single-arm and multi-arm, clinical trials. Studies including individual case reports, letters, single-arm studies, case-control studies, reviews, studies reporting other diseases than AML, and non-human research were excluded. The following characteristics were extracted: the first author’s name, publication time, condition, age, sample size, clinical trial ID, sex, outcome measures and treatments.

### 2.3 Outcomes

The primary endpoint was overall response rate Response (Overall Response Rate (ORR), Complete Response (CR), Complete response with incomplete count recovery (CRi). The secondary endpoint were survival (Overall Survival (OS)and adverse effects> grade 3 (immune-mediated AEs).

### 2.4 Statistical Analysis

As described in the guide of Muka et al., after systematic review is run and enough evidence found, a meta-analysis should be performed (12). As there were different clinical trials included, the clinical heterogeneity was analyzed using a random effects model. Statistical significance was determined with the Cochran’s chi square test. The heterogeneity of the studies was calculated with the Tau and I2 tests. A p value of <0.05 was considered statistically significant. The heterogeneity was assessed using I2 values: low (I2 = 0%–25%), medium (I2 = 25%–50%), high (I2 = 50%–75%), and nonignorable (I2 = 75%–100%). The subgroup and sensitivity analyses were conducted to analyze the heterogeneity among studies.

## 3 Results

### 3.1 Selection of Studies Search Results

**Figure 2:**
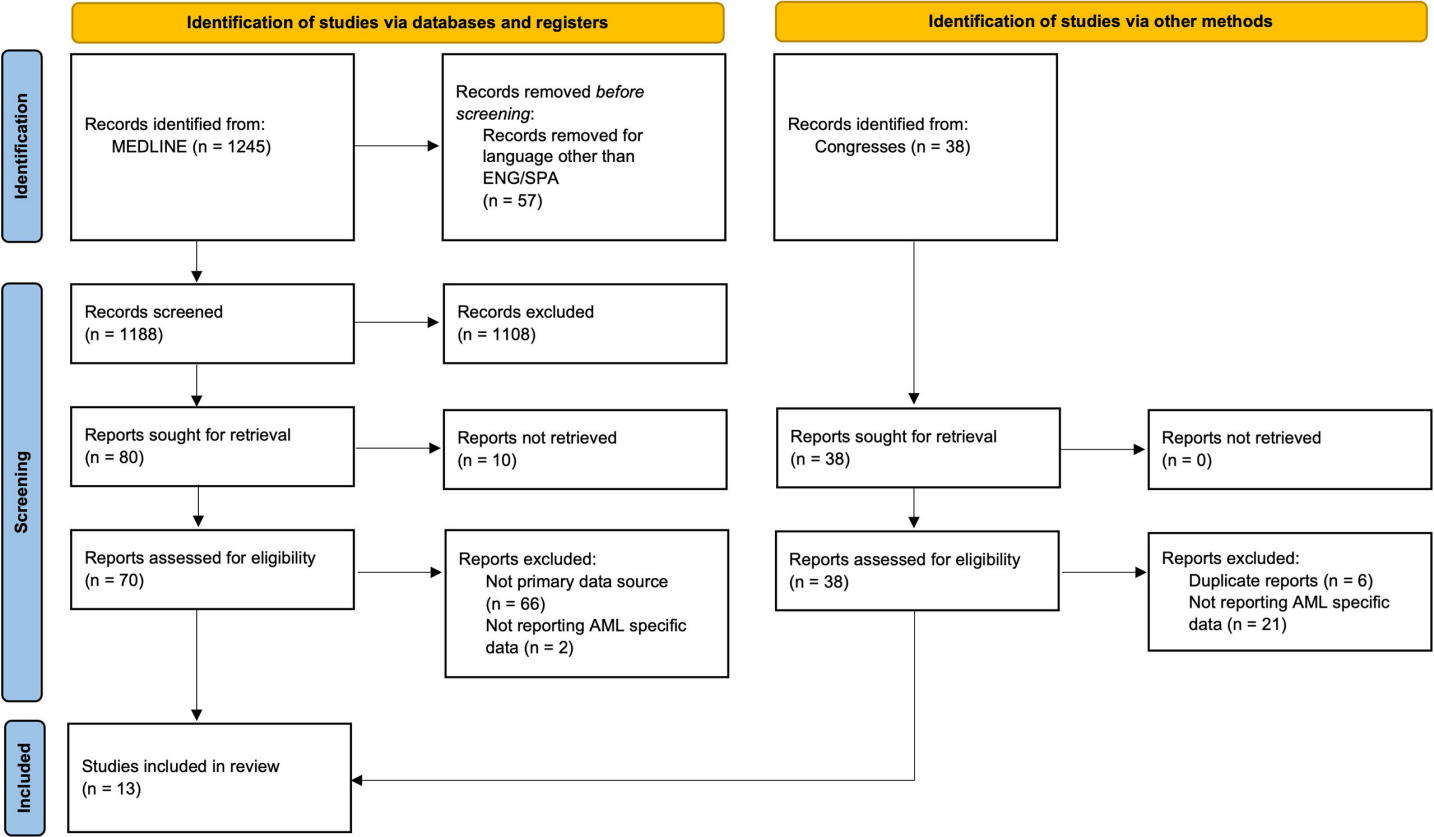
Flow Diagram of included studies. Diagram of search results with ASReview, detailing the steps of the systematic review.

A total of 1,245 publications were identified within the PubMed search and were assessed with the help of ASReview to find relevant publications. From those publications, there were 80 abstracts that matched our selection criteria. This meta-analysis was conducted in accordance with the Cochrane Handbook for Systematic Reviews, Preferred Reporting Items for Systematic Review and Meta-Analysis (PRISMA) statement (13). After removing duplicated studies *via* Mendeley and screening the titles, abstracts, and full texts of all eligible studies, we used the following standards to select studies for inclusion: (1) clinical studies involving controlled trials with samples >5 cases, case reports, letters, and reviews were excluded; (2) sufficient data on efficacy and adverse events (AEs). Most of the identified trials in the systematic review were early phase trials. The detailed search flow is displayed in **Figure 2**.

#### 3.1.1 Eligible Studies and Characteristics

A total of 13 studies was identified as relevant publications to be included in the meta-analysis. The vast majority of them were early phase trials (including phase I, IB and phase II), which mainly measured these immunotherapies’ toxicity profiles and response rates (11, 14–25). The median age of the patients among the studies was 71. In terms of criteria inclusion, there were included *de novo* AML or secondary AML, and different lines of treatment, first line and R/R. Finally, the cytogenetic risk profile was also explored, and it is relevant to highlight that among the included studies in the meta-analysis, the adverse cytogenetic risk profile was around 50%.

### 3.2 Overall Response Rate (ORR)

**Figure 3:**
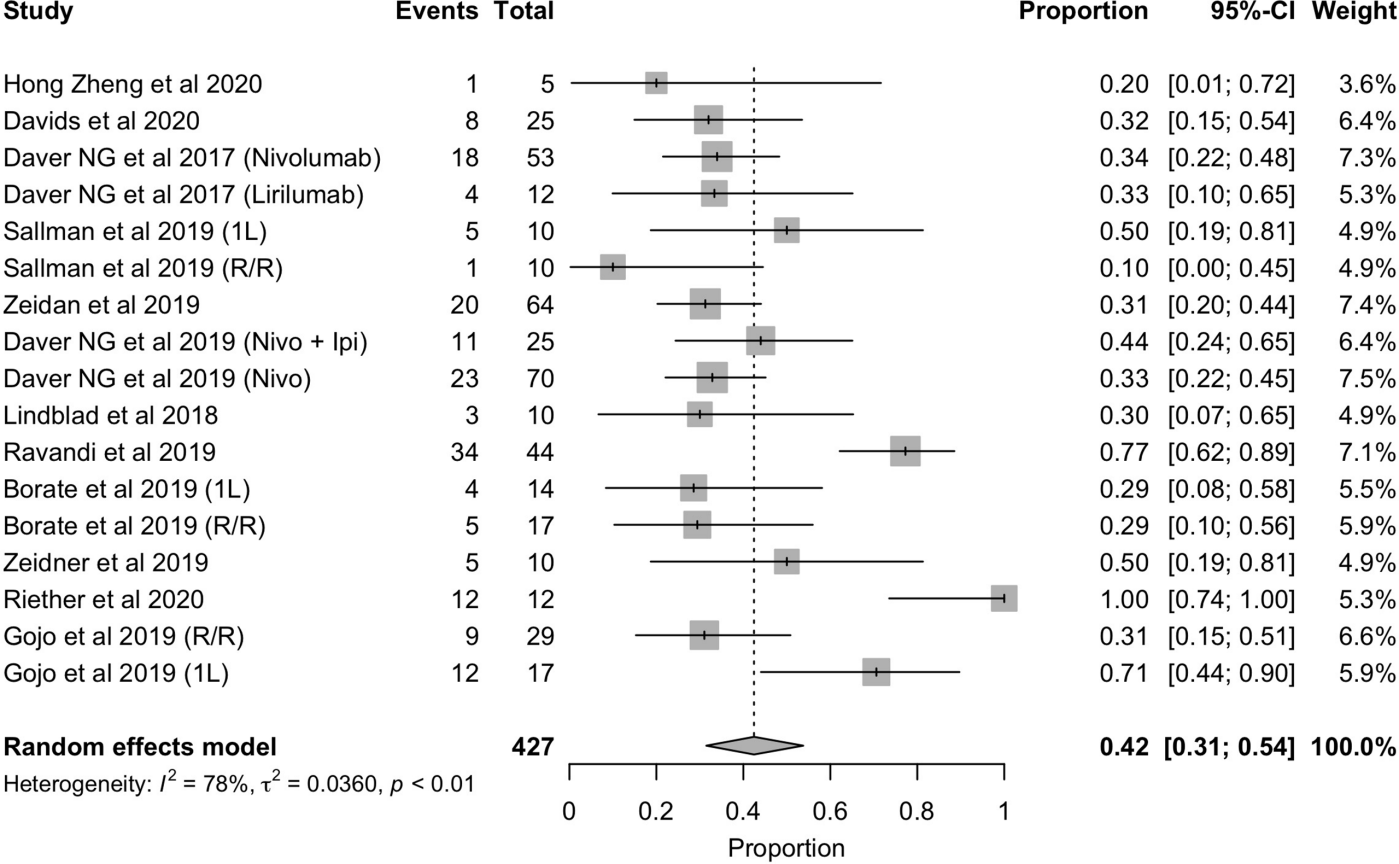
Forest plot of overall response rate in patients with AML.

**Figure 4:**
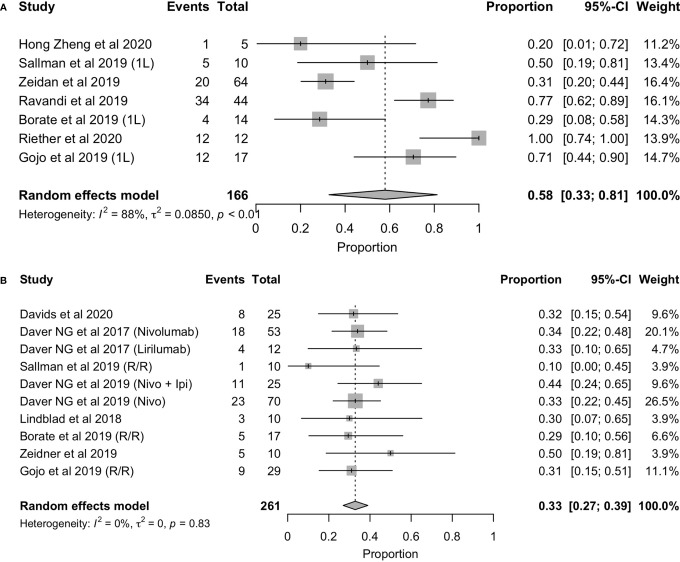
Forest plot of overall response rate in patients with first-line **(A)**, refractory/relapsed AML **(B)**.

In terms of response rate, the overall response rate with these immunotherapies was 42% (**Figure 3** **)**. However, as it has been observed with other therapies approved for the treatment of AML, it is necessary to distinguish between those patients who are treated in first line (1L) and those who have relapsed or who are refractory to prior therapy (R/R) and are receiving advanced lines. For this reason, we analyzed the overall response rate in each of these scenarios, the results of which are shown in **Figure 2**. As expected, the overall response rate is higher with immunotherapy in the first-line setting (ORR= 58%, 95%CI: 33% - 81%) than in the relapsed and/or refractory setting (ORR = 33%, 95%CI: 27% - 39%) (**Figures 4A, B** **)**.While these rates may indicate the efficacy of these therapies in the treatment of AML, it should not be overlooked that the vast majority of these studies used these approaches in combination with chemotherapy and/or allo-HSCT. Therefore, it cannot be concluded that the high first-line response rate should be attributed to the use of these new therapeutic options.

### 3.3 Complete Response Rate With or Without Hematologic Recovery (CR/CRi)

**Figure 5:**
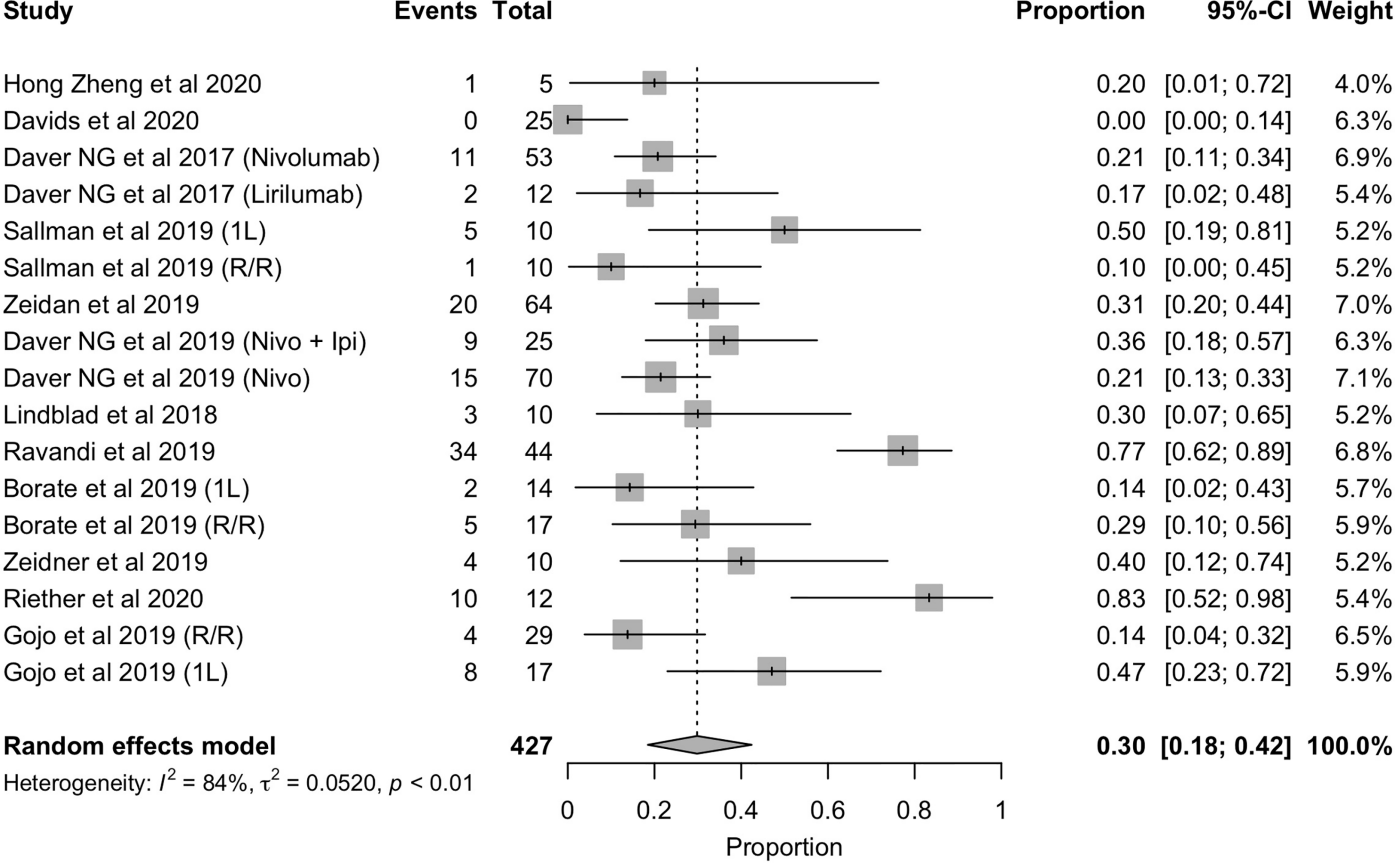
Forest plot of CR/CRi in patients with AML.

**Figure 6:**
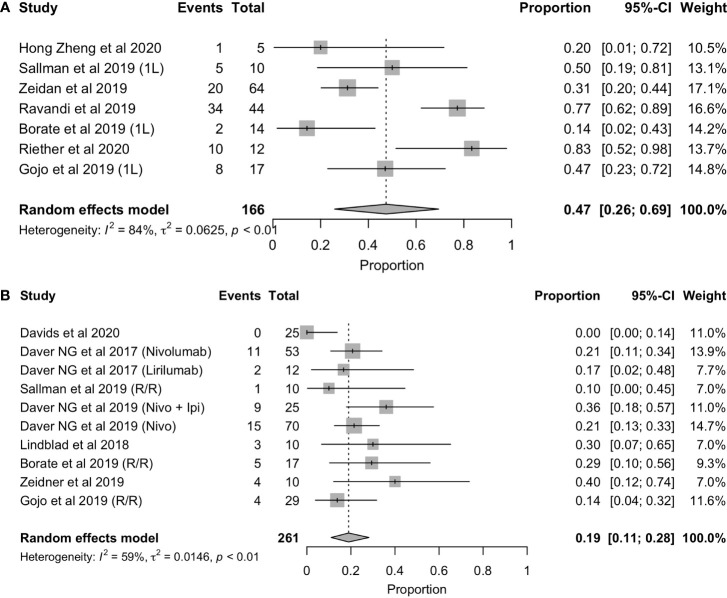
Forest plot of overall response rate in patients with first-line **(A)** and refractory/relapsed **(B)** AML.

With regard to complete response rates, global result was 33% (CRR=33%,95%CI: 22%-45%) (**Figure 5** **),** regardless of treatment line. Nevertheless, we observed statistically significant differences between the complete response rate (CR/CRi) in patients treated first-line and those in relapse and/or refractory, 47% (95%CI: 26%-69%) and 19% (95%CI: 11%-28%), respectively (**Figures 6A, B** **).** However, it is essential to highlight the disparity among the results of the different first-line trials, some of which were very positive and some others showed a very limited efficacy. As with the overall response rate, these results mostly reflect the effect of these new options in combination with other therapies and/or allo-HSCT, which distorts the interpretation of these results.

### 3.4 Overall Survival (OS)

Due to the lack of consistency in the reporting of this variable across the trials, in this section we have proceeded to calculate the mean and median of these results, without being able to represent the forest plot of these results, due to the limitation of evidence. Despite the small number of studies included (13 trials), the average of overall survival was 8.9 months, with a median of 8.0 months (95%CI: 3.9 - 15.5 months). Differences between first line and R/R settings were observed, since the average of overall survival in first line was (n = 4) 12.0 months, with a median of 13.1 months (95%CI: 4.7 - 17.7 months), duplicating the OS in R/R of 7.3 months (mean), and median of 6.7 months (95%CI: 4.5 - 10.7 months).

Despite the lack of published evidence about this variable, we noted a greater overall survival in the studies in which patients were treated in first line with immunotherapy than in those in which treated patients were in relapse or were refractory to a first line. However, in the absence of a phase III clinical trial that directly compares the effect of these new therapies vs. current treatment standards, it cannot be concluded that these therapies prolong survival.

### 3.5 Immune-Mediated Adverse Effects (AEs)

**Figure 7:**
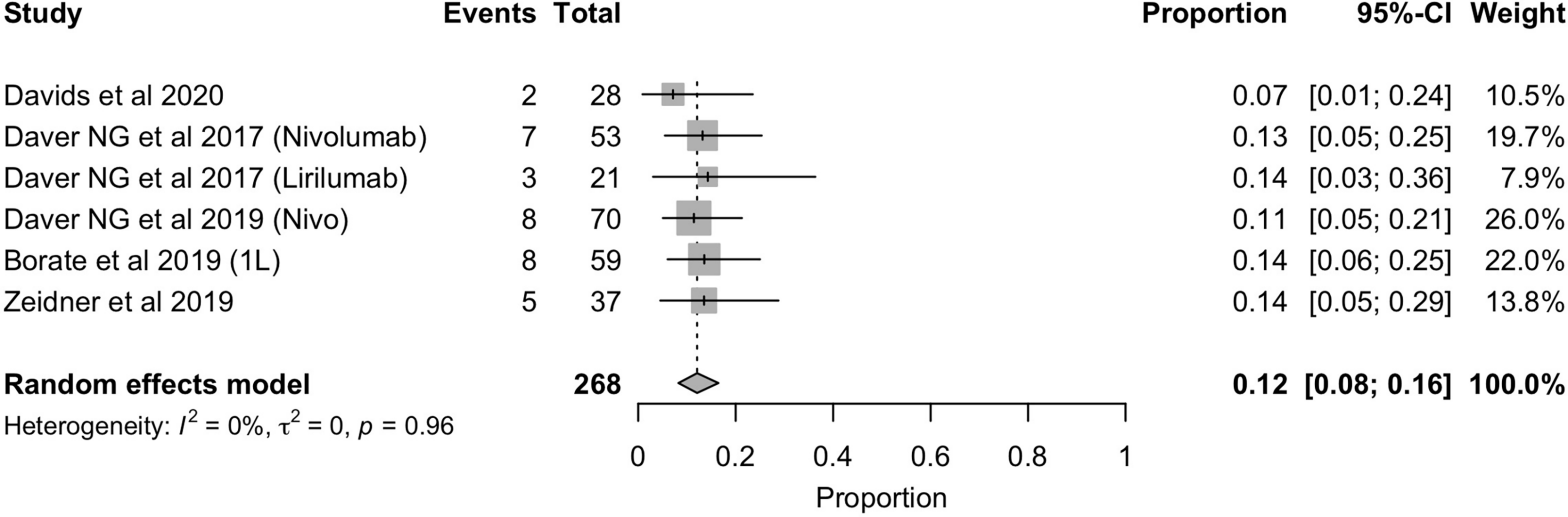
Forest plot of immunomediated AEs in AML patients.

In terms of toxicity, it was also registered the immune-mediated AEs that are directly related to the use of these therapies. The frequency of immune-mediated AEs, equal to or greater than toxicity grade 3, was 12% (95%CI: 8% - 16%) and was homogeneus among the identified studies (**Figure 7**). The toxicity profile of these therapies is widely known in other scenarios (solid tumors and other hematological malignancies) and is relatively better than that of chemotherapy used for the treatment of AML.

### 3.6 Publication Bias and Sensitivity Analysis

Publication bias is a matter of concern in meta-analysis. In this meta-analysis, a funnel plot of the 13 studies was built in order to study the publication bias. Results suggest the presence of publication bias. (**Supplementary Material Figure 2**). As it can be observed, an asymmetrical plot indicates the presence of publication bias due to low methodological quality of smaller studies. Inferior areas, both left and right, indicates small studies of inadequate quality whose results are biased towards larger beneficial effects. We had no other option but to assume this limitation due to the lack of larger and more advanced clinical trials.

## 4 Discussion

The complete response rates of the first-line young patients receiving high-dose chemotherapy with cytarabine and anthracycline are in the range of 50%-60% on intermediate risk disease and up to 80% on favourable risk disease, while in adverse risk disease is just around 40%. In the latter case, even followed by allogeneic hematopoietic stem cell transplantation (Allo-HSCT), half of these patients relapse. Besides that, the combination of chemotherapy and new targeted drugs such as FLT3 inhibitors, particularly the addition of midostaurin, increases moderately the overall survival. Furthermore, the addition of monoclonal antibody anti-CD33 drug conjugate (gemtuzumab-ozogamicin) also increases the overall survival and decreases the risk of relapse in favourable and intermediate risk. On the other hand, treatment with HMA in patients unfit for intensive chemotherapy have a CR rate of 28%. It is important to highlight that the combination of BCL-2 inhibitor (venetoclax) with an HMA has registered better results than the standard of care in patients with adverse risk disease. Recently, the combination of HMA and venetoclax in patients with adverse-risk cytogenetics and those at least 75 years old showed overall response rates of 60% and 65%, respectively (26).

In this context, the experience with these treatments in the field is still limited, and the results provided by this metanalysis are modest according to the rates mentioned before (ORR = 42% and CR/CRi = 33%) (**Figures 3** **,** **5**). Nevertheless, the majority of studies included are still early trials, so, further investigation is required.

Nonetheless, it is important to consider the treatment line to assess the ICI efficacy profile. While patients with R/R AML might present lower response rates, newly diagnosed cases typically exhibit higher response rates. Although this tendency can also be found with approved therapies, the exhaustion of immune cells is probably increased in the R/R setting, limiting the potential efficacy of immunotherapy. In this review, we have seen another prove of this rationale when comparing ORR (62% vs. 33% for first line and R/R) (**Figure 4**).

This trend is not only seen when assessing ORR but also with CR/CRi (51% vs. 22%) between both settings (**Figure 6**). Gojo et al. reported a 14% CR/CRi in the R/R setting and a 47% CR/CRi in newly diagnosed patients using pembrolizumab, a PD-1 inhibitor, in combination with 5-azacytidine. Superior efficacy of ICI in first-line treated patients compared to the relapsed/refractory line was observed, which could be in line with a better prognosis of patients treated in earlier lines. It also could be related to the difference between first-line vs R/R of AZA monotherapy in terms of CR rates (28% vs 15%) and median OS (10.4 months vs 8.4 months) (27). Another explanation could be related to the increased immune cell count and fitness in first-line patients. In this line, Sallman et al. (23) described an example of beneficial effect of ICI in early lines by testing the combination of Hu5F9-G4 (5F9), an antibody targeting CD47, with azacytidine. In terms of response, a 50% CR/CRi was reported in newly diagnosed AML patients, while this rate was a 10% in R/R AML (23). The mechanism of tumor phagocytosis was induced by the blockade of CD47, and the addition of 5-Azacytidine could synergize with the effect of phagocytosis (28). On the contrary, Borate et al. (24) reported no differences in overall response rate between first-line and R/R patients when using MBG453, a TIM-3 inhibitor, in combination with decitabine (29% both groups). Furthermore, it was reported a CR/CRi in R/R that doubled the first-line rate (29% vs. 14%). These results, although controversial, may suggest TIM-3 as a therapeutic target, especially in the R/R, and support further clinical trials of MBG453 in combination with HMAs in patients with AML (24).

While most of these response rates may represent an indication of the efficacy of these therapies in the treatment of AML, it should not be overlooked that the vast majority of these studies used these therapies in combination with chemotherapy and/or allo-HST. Therefore, the high first-line response rates cannot be entirely attributed to the use of these new therapeutic options. In this review, the most common combinations were HMA and PD-1 blockers, CTLA-4, CD47, TIM-3, CD70 or KIR, due to the expected synergistic effect beforementioned.

Regarding the immunotherapy’s mechanism of action, efficacy has been found to be directly related to tumor mutational burden and neoantigens formation (29). As reported by Castle et al. AML shows one of the lowest tumor mutational burdens across oncologic conditions (30). Consequently, it provides possible justification as to why the response rates to these treatments are so limited in AML. As relatively few mutations take place in AML patients, low levels of neoantigens are generated, preventing these patients from benefiting from ICI therapy. In other words, high tumor mutational burden can guide response to immunotherapy treatment. To successfully use this kind of therapies, standardized biomarkers are needed to help predict response and facilitate the selection of patients who may benefit from its use (31). Also, as new checkpoint inhibitors emerge, the need to further understand the efficacy profile of these approaches increases. Relationships between ICI mechanisms of action and genetic profiles may provide further information as where and when to use these therapies (32).

Toxicity related to these therapies must also be cautiously analyzed. The most common adverse events in patients with AML, reported in the included clinical trials, are hematological adverse events (AEs), including moderate-severe thrombocytopenia, febrile neutropenia and severe anemia. Nonetheless, the most frequent hematological AEs were related to non-severe cytopenia. Particularly, the most specific AEs of these therapies are immune related adverse events (irAEs), derived from their inflammatory effects, including damages to skin, gastrointestinal, hepatic, endocrine and pulmonary tissues. Grade ≥3 irAEs rate was similar among studies [12% (95%CI: 8% - 16%)] with transaminitis and maculopapular rash being the most common adverse events (**Figure 7**). Although transaminitis, skin rash and pneumonitis accounted for 46%, 42% and 15%, respectively, some unusual adverse events were identified, such as colitis, arthritis and nephritis. No events of death related to immunotherapy were registered in the studies included in this review. Davids et al. (18) reported that the only factor associated with the development of irAEs was ECOG performance status. Nevertheless, due to the small number of patients who suffered irAEs, further studies are needed to establish this relationship.

## 5 Conclusion

Immunotherapy based on checkpoint inhibitors in combination with intensive chemotherapy, hypomethylating agents or other targeted therapies is gaining interest in the management of hematological malignancies such as AML. However, results obtained from clinical trials are modest and limited by both, type of design and clinical trial phase. Hopefully, prospective study of responses to this type of treatments according to different biological profiles might provide strategies to identify those patients who may benefit from ICI.

Nonetheless, it is important to highlight the limitations of this review where most of the studies were early phase trials. High heterogeneity was found among the studies and no subgroup analyses according to relevant prognostic factors, such as cytogenetics risk profile, was performed. Also, most of included studies were reported as congress abstracts, potentially missing relevant information that could affect this meta-analysis results. Thus, further investigation is required to clarify profile of patients most likely to derive clinical benefit. Looking ahead, phase III randomized clinical trials are needed to comparatively study the efficacy of these agents, either directly against standard treatments or in combination with them.

## Data Availability Statement

The original contributions presented in the study are included in the article/**Supplementary Material**. Further inquiries can be directed to the corresponding author.

## Author Contributions

MG-L participated in the data collection, data review, relevant data extraction, data analysis, statistical analysis, and the writing of the manuscript. JCM participated in checking relevant data extraction as well as in the statistical analysis and the writing of the manuscript. APR and IGC participated in checking data extraction as well as in the data analysis, and the writing of the manuscript. AMO, substantial contributions, and manuscript review. All authors saw and approved the final version.

## Conflict of Interest

Author JCM was employed by A&P Lifescience Advisors.

The remaining authors declare that the research was conducted in the absence of any commercial or financial relationships that could be construed as a potential conflict of interest.

## Publisher’s Note

All claims expressed in this article are solely those of the authors and do not necessarily represent those of their affiliated organizations, or those of the publisher, the editors and the reviewers. Any product that may be evaluated in this article, or claim that may be made by its manufacturer, is not guaranteed or endorsed by the publisher.

## Supplementary Material

Supplementary Figure 1 PICOS scheme.

Supplementary Figure 2 The sensitivity analyses of ORR in data from 13 trials of patients with AML.

Supplementary Figure 3 Overview of randomized clinical trial of ICI on AML.

Supplementary Figure 4 Summary of results included in the meta-analysis.
