# Supplementary figures and images for: Immune Checkpoint Inhibitors in Acute Myeloid Leukemia: A Meta-Analysis
Source: Front Oncol. 2022 Apr 21;12:882531. doi: 10.3389/fonc.2022.882531 (PMC9069679; doi:10.3389/fonc.2022.882531)

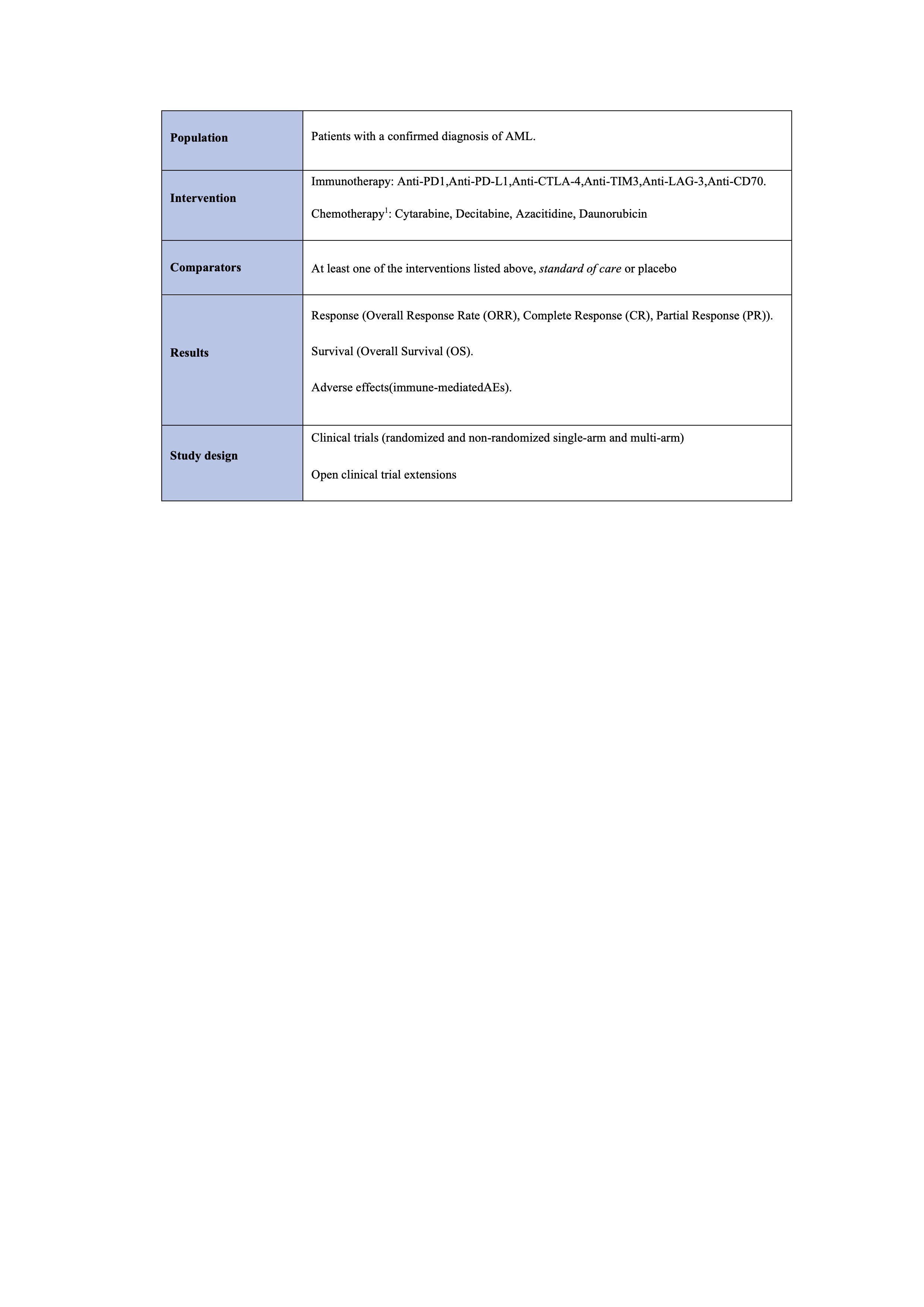

Supplement: Supplementary Figure 1 — PICOS scheme. [file Image_1.jpeg]

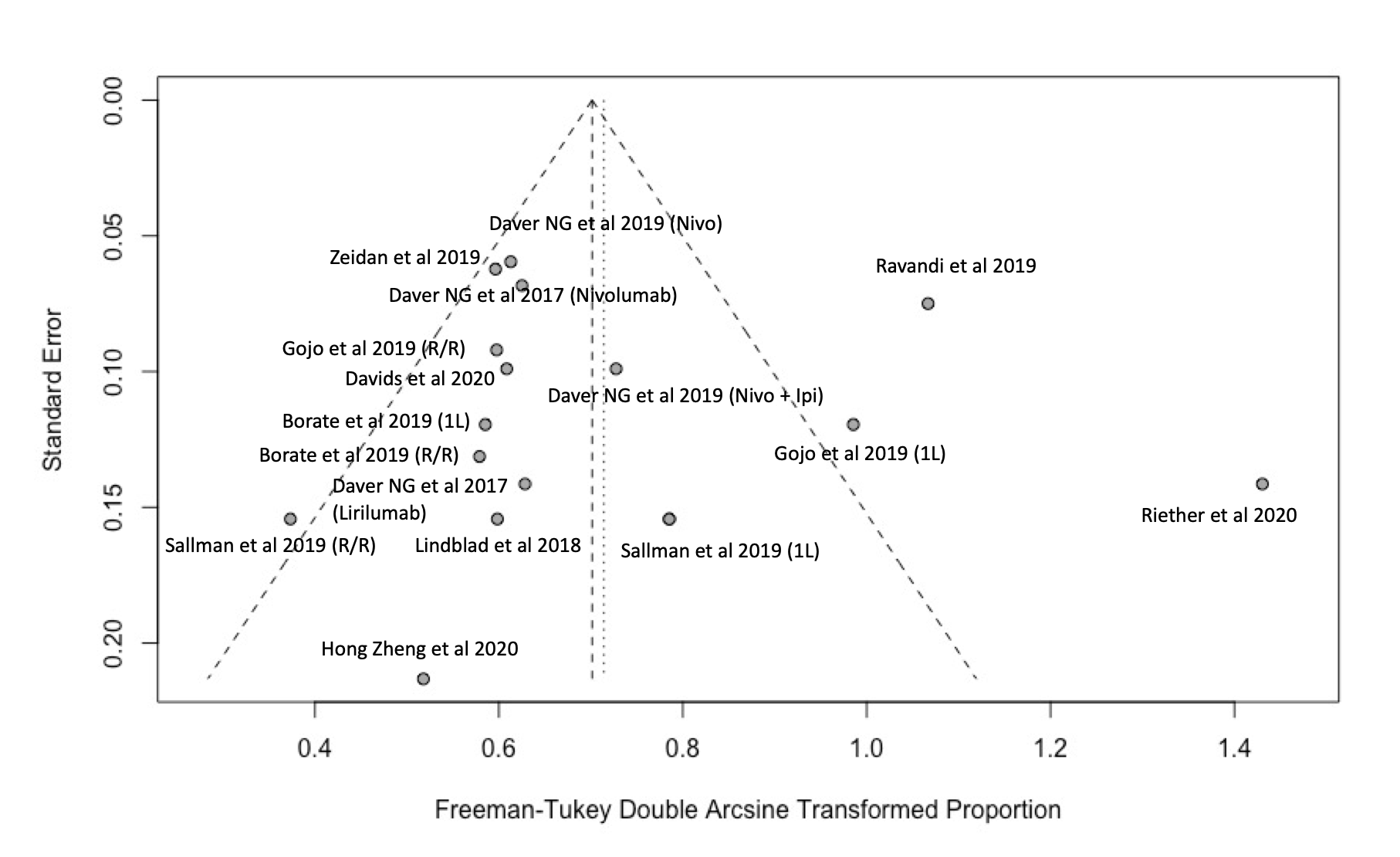

Supplement: Supplementary Figure 2 — The sensitivity analyses of ORR in data from 13 trials of patients with AML. [file Image_2.png]

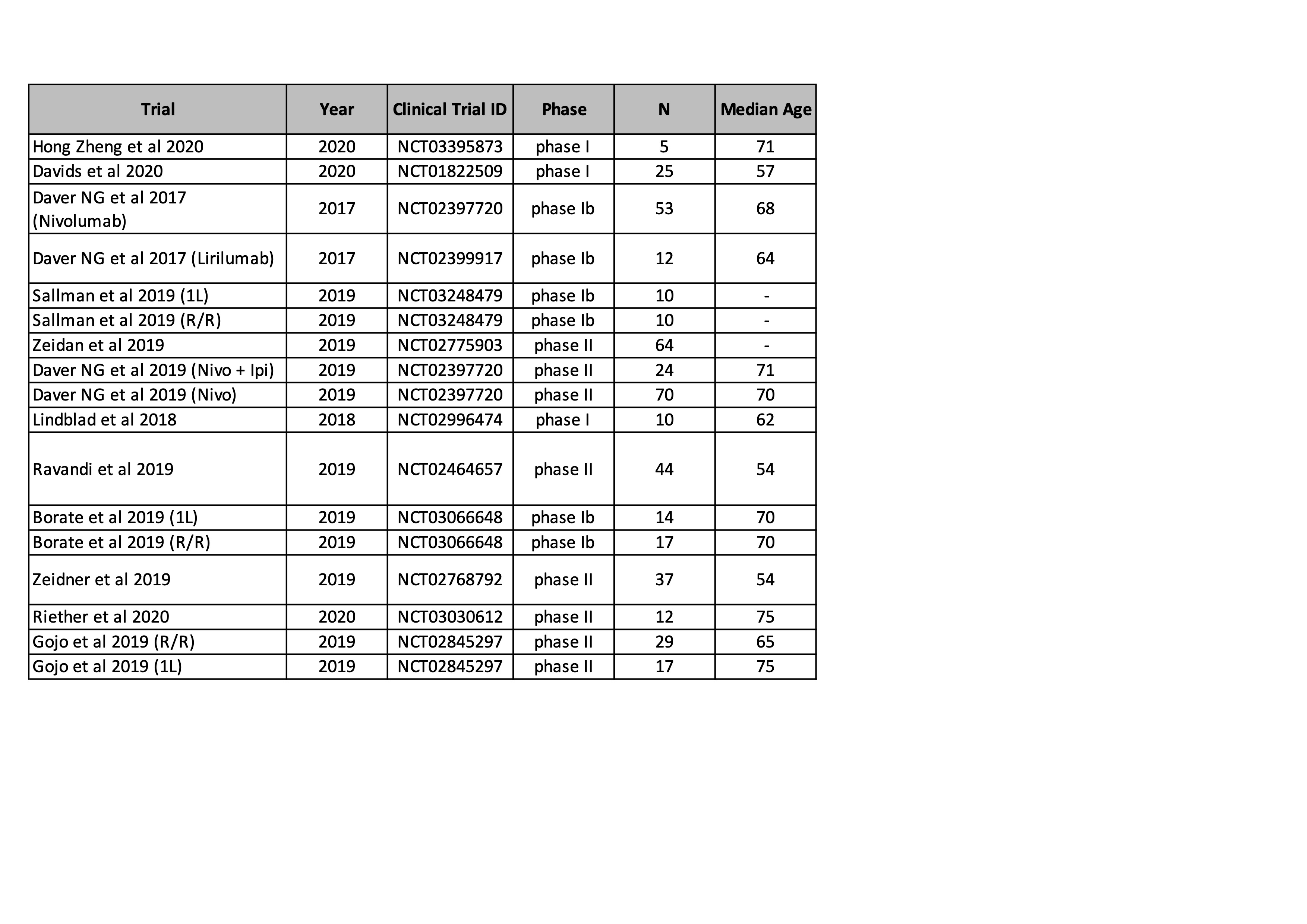

Supplement: Supplementary Figure 3 — Overview of randomized clinical trial of ICI on AML. [file Image_3.jpeg]

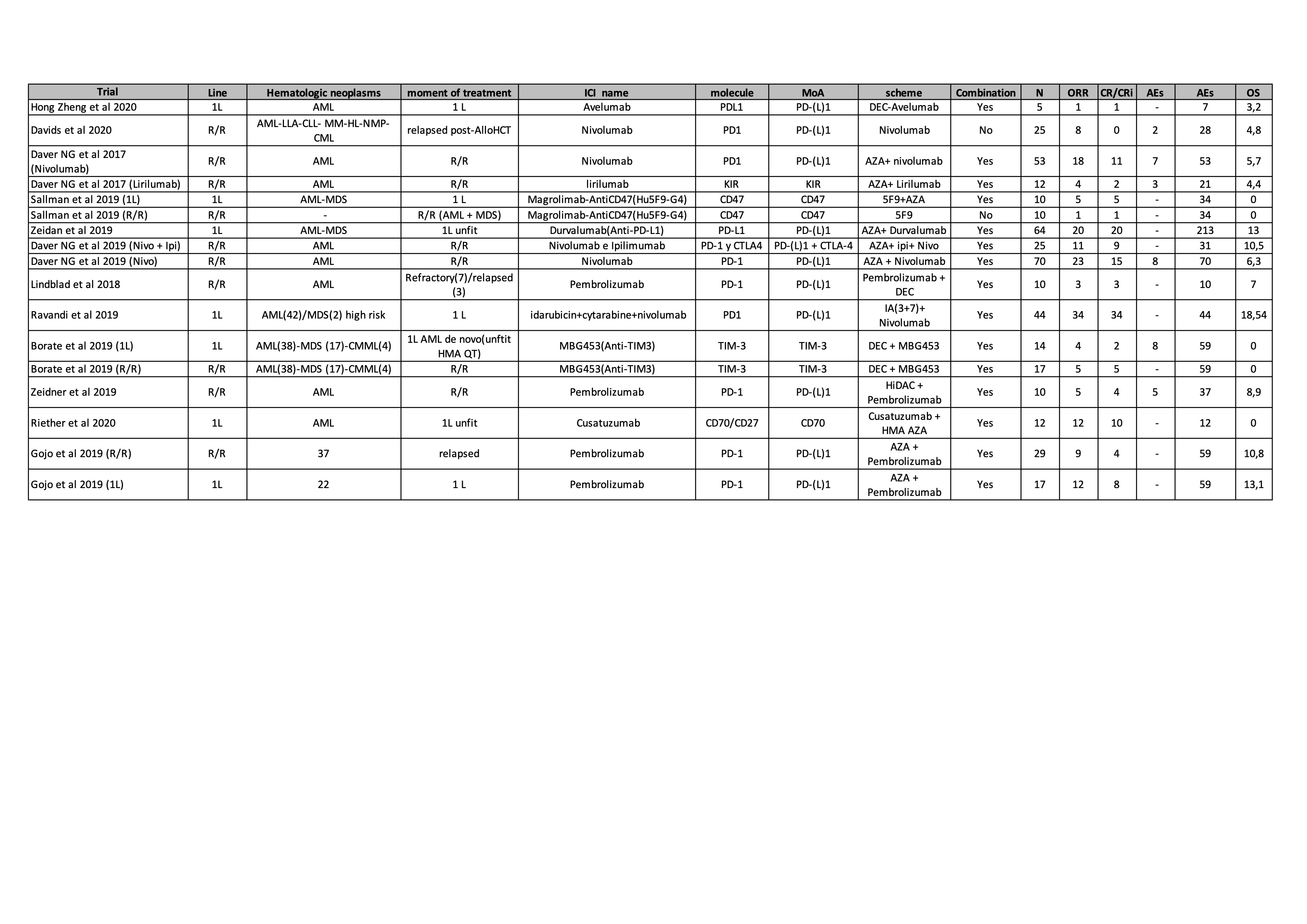

Supplement: Supplementary Figure 4 — Summary of results included in the meta-analysis. [file Image_4.jpeg]
